# Hastening Progress in *Cyclospora* Requires Studying *Eimeria* Surrogates

**DOI:** 10.3390/microorganisms10101977

**Published:** 2022-10-06

**Authors:** Matthew S. Tucker, Asis Khan, Mark C. Jenkins, Jitender P. Dubey, Benjamin M. Rosenthal

**Affiliations:** Animal Parasitic Disease Laboratory, Beltsville Agricultural Research Center, Agricultural Research Service, United States Department of Agriculture, BARC-East, Beltsville, MD 20705, USA

**Keywords:** *Eimeria*, *Cyclospora*, coccidia, surrogates, detection, control, oocysts, viability, sand filtration, diagnostics

## Abstract

*Cyclospora cayetanensis* is an enigmatic human parasite that sickens thousands of people worldwide. The scarcity of research material and lack of any animal model or cell culture system slows research, denying the produce industry, epidemiologists, and regulatory agencies of tools that might aid diagnosis, risk assessment, and risk abatement. Fortunately, related species offer a strong foundation when used as surrogates to study parasites of this type. Species of *Eimeria* lend themselves especially well as surrogates for *C. cayetanensis.* Those *Eimeria* that infect poultry can be produced in abundance, share many biological features with *Cyclospora*, pose no risk to the health of researchers, and can be studied in their natural hosts. Here, we overview the actual and potential uses of such surrogates to advance understanding of *C. cayetanensis* biology, diagnostics, control, and genomics, focusing on opportunities to improve prevention, surveillance, risk assessment, and risk reduction. Studying *Eimeria* surrogates accelerates progress, closing important research gaps and refining promising tools for producers and food safety regulators to monitor and ameliorate the food safety risks imposed by this emerging, enigmatic parasite.

## 1. *Cyclospora*: Simple to Understand, but Difficult to Study

*Cyclospora cayetanensis* is a human coccidian parasite with a one-host, fecal–oral life cycle. The parasite (reviewed in [1]) causes severe illness worldwide in developed and developing countries. It currently lacks *in vitro* and animal models, making it challenging to study. Clearly, there is a need to advance research on this enigmatic parasite that could address food safety, epidemiology, detection, and other current needs. How much can produce growers, packers, and retailers do to minimize the public health burden, reputational harm, and expense posed by *Cyclospora*? One prominent produce safety professional recently opined that “*Cyclospora* may not be our fault, but it is our problem.” Produce growers, packers, and retailers understand that outbreaks of enteric disease risk costly product recalls, imperil public health, and undermine public confidence. However, available data fail to firmly establish what steps hold greatest promise to mitigate these risks and costs, or the extent to which improvements to produce safety will reduce the public health burden. Here, we review present obstacles and opportunities to achieving answers, emphasizing opportunities to clear longstanding barriers imposed by the scarcity of *Cyclospora* oocysts by studying surrogate organisms that can be generated by the millions and studied in their natural hosts.

In concept, reducing human exposure could not be simpler: reduce human fecal contamination in water and food. Success does not require managing wildlife, insect vectors, or other intermediaries. Further simplifying the task, these parasites do not reproduce in fields, food processing facilities, in transit, in the produce case, in restaurants, or foods purchased (whether refrigerated or not). Once excreted, their numbers do not increase.

Unfortunately, these parasites need not further multiply to pose a public health risk, and outbreaks continue. Oocysts, once sporulated, endure environmental extremes. Furthermore, just a few suffice to establish infection. Transmission exploits a long-established mode for parasites of this kind: persist and exploit the host’s need to eat or drink. For industry and regulators, this presents a “needle in the haystack” problem, and current diagnostic tools fail to provide industry, researchers, or regulators sufficient:

- Specificity.
- Speed.
- Cost effectiveness.
- Means to assess parasite viability.

## 2. The Scarcity of Oocysts Slows Research Progress

Researchers face daunting challenges when seeking to blunt parasite’s deleterious public health and economic impact because infected people constitute the principal source of research material. To date, no attempts to propagate *C. cayetanensis* in other animals, or in cell culture, have succeeded. Indeed, an attempt by researchers to voluntarily infect themselves failed [2]. Ethical barriers appropriately strictly limit such attempts.

Despite such limitations, researchers studying limited supplies of *C. cayetanensis* have made commendable progress characterizing strains, genomes, and advancing detection. Several *C. cayetanensis* draft genomes have been characterized and published [3,4,5,6,7,8,9] and molecular typing efforts have given rise to powerful genotyping tools [10,11,12,13,14,15,16,17,18,19,20,21] for epidemiological analysis, such as outbreak tracing. As described below, methods such as these and others are now being used to detect *C. cayetanensis* from various sources of infection.

Advances in organoid culture [22] and transgenic mice [23] offer hope that human-derived oocysts might someday become a renewable resource. These resources have been used with success to study the related coccidians *Cryptosporidium* [24,25] and *Toxoplasma* [26,27,28], and the distantly related apicomplexan *Plasmodium* [29,30,31]. However, at present, supplies remain preciously limited; clinics in endemic areas willing and able to test patients experiencing enteric disease represent the most reliable source of research material. However, clinicians encountering symptomatic individuals may easily miss the opportunity to collect oocysts in large numbers and may lack the expertise or interest necessary to preserve oocysts for future use as research specimens. Clinicians participating in outbreak responses account for most of the materials available for scientific study.

Then, too, researchers fortunate enough to access these parasites face daunting limits as to the range of questions they can study. Oocysts downregulate metabolic activity, conserving resources until ingested by a susceptible person. Cell culture systems might allow researchers to glean valuable information about this parasite’s vulnerabilities, but progress will require a stroke of luck and an abundance of oocysts.

Such obstacles slow the progress of research teams willing to risk disappointment and delays, and undoubtedly dissuade enterprising researchers from seeking to build a career studying this enigmatic parasite. Among the United States (U.S.). Government agencies, the Food and Drug Administration (FDA) and the Centers for Disease Conteol and Prevention (CDC) have committed significant resources to developing diagnostic tools and markers suitable for outbreak tracing (cited in this work). The National Institutes of Health (NIH) has refrained from making large investments in this arena. The U.S. Department of Agriculture (USDA) supports a few in-house projects (at the Agricultural Research Service [ARS], including our team) and the National Institute of Food and Agriculture (NIFA)-USDA has sought coordinated research in this area. That would represent a major public investment for academic researchers that have instead relied, to a great extent, on produce industry investments necessarily limited in scope and duration.

To hasten progress on some of these fronts, we advocate exploiting appropriate, abundant surrogate organisms, for reasons detailed below. These surrogates should, for example, accelerate efforts to establish:

- Filtration systems to remove oocysts from irrigation or water in processing plants.
- Detection.
- Sanitizing systems, including chemical and physical treatments, for fresh produce and food processing equipment.
- Cheaper, faster surveillance tools.
- More specific and sensitive diagnostic tools.
- Viability assays, to better determine food safety risk.

## 3. Suitable Animal Models Hasten Progress

### 3.1. Researchers Presently Lack a Species of Cyclospora That Cycles in an Established Laboratory animal Model

Certain species of *Cyclospora* infect non-human primates [32,33,34,35,36,37]. Available evidence suggests that each species infects exclusively one host species, but the extent of heterologous transmission remains poorly defined. *Cyclospora macacae*, the most recently described species, infects rhesus monkeys (*Macaca mulatta*) [38]. *Cyclospora* oocysts in chimpanzees (*Pan troglodytes*) and Cynomolgus monkeys (*Macaca fascicularis*) resemble *C. cayetanensis* at the Internal Transcribed Spacer-2 (ITS-2) portion of ribosomal DNA (rDNA) [39]. However, for ethical and practical constraints severely constrain their use as research subjects. The host specificity of each parasite remains unclear, warranting caution when assigning species status [39]. Attempts to infect numerous animals with *C. cayetanensis* have failed, suggesting host specificity [40]; clinicians attribute all human infections to *C. cayetanensis*. However, experimental infections of human volunteers can fail to produce symptoms or oocysts, suggesting variability in host susceptibility and/or oocyst infectiousness [2].

Oocysts resembling *Cyclospora* occur in the feces of dogs, mice, rats, monkeys, ducks, chickens, and other birds [41,42,43,44,45,46]. Conceivably, their presence might merely support passage through the gastrointestinal tract (with no reproduction in host cells) [47]. Molecular methods identified oocysts of *Cyclospora* spp. in dairy cattle feces [48]. Molecular detection confirmed microscopy documenting oocysts in dogs, chickens, and rhesus monkeys (*M.mulatta*) [49]. *Cyclospora*-like oocysts occur in the feces of several other species, including carnivores, artiodactyla, and nonhuman primates from a zoological garden from Spain [44] by coprological analysis; no molecular methods or sequencing were then performed. Debate surrounds any contribution that free-living nematodes, insects, and rotifers may make to disseminating *Cyclospora* [50]. Historically, *Cyclospora* were considered parasites of moles [51]. The only rodent species named is *Cyclospora angimurinensis*; it was found in 1 of 20 pocket mice (*Chaetodipus hispidus*) from Texas [52]. Whether it is a true parasite or will infect the common house mouse (*Mus musculus*) is unknown.

### 3.2. Eimeria That Infect Poultry Provide Especially Useful Surrogates for C. cayetanensis

**Figure 1:**
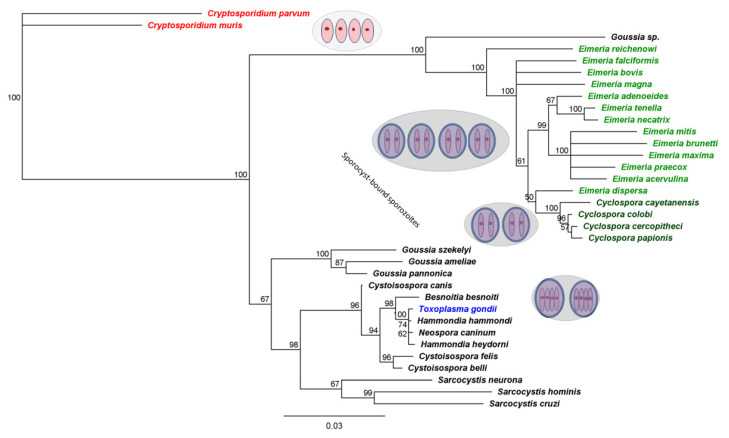
Phylogenetic affinity of *Cyclospora spp.* to *Eimeria spp*. *Cyclospora* and *Eimeria* (green). Tissue-cyst forming coccidians such as *Toxoplasma gondii* (blue), also encapsulate their sporozoites in environmentally resistant sporocysts. More distantly related parasites, such as *Cryptosporidium,* lack sporocysts. This tree was reconstructed on 100 bootstrap replicates using the Neighbor-Joining method as implemented in Geneious Prime (Biomatters); the inference of *Cyclospora* as nested within *Eimeria* withstands a variety of plausible evolutionary models and reconstruction methods, e.g., [53,54,55,58].

*C. cayetanensis* is a member of a large, ubiquitous family of coccidian parasites in the phylum Apicomplexa. This broad assemblage of eukaryotic, intracellular parasites includes vector-borne agents (such as those that cause malaria) and those that cycle between predator and prey (such as the agent of toxoplasmosis). Coccidian parasites are transmitted among members of a single host species, via fecal–oral transmission. Such parasites are ubiquitous among vertebrate hosts, having initially evolved as parasites of fish and then diversifying with every terrestrial vertebrate group [53]. Unsurprisingly, the closest known relatives of *C. cayetanensis* are congeners that infect non-human primates. Knowing this may advance efforts to establish cell culture in human or primate cell lines; but this fact practically precludes establishing an animal model for this pathogen because primates do not represent suitable experimental subjects except under the most extreme of circumstances. However, the very first studies of *Cyclospora* identified evolutionary affinity to species of *Eimeria*, which cause similar enteric infections in a vast range of vertebrate hosts [54,55] (Figure 1).

Hundreds of *Eimeria* spp. have been described. Among these, parasites closely related to *Cyclospora* are pathogens of poultry: seven broadly recognized *Eimeria* species infect chickens, alone [56]. The close similarity of *Eimeria* to *Cyclospora* established by phylogenetic reconstruction (Figure 1) shows that *C. cayetanensis* are especially closely related to *Eimeria* that infect poultry [1,3,5,57,58]. Indeed, these species of *Eimeria* appear more closely related to *Cyclospora* than to other species ascribed to the genus *Eimeria.* Such similarity explains other biological and biochemical similarities. For example, *Eimeria* and *Cyclospora* synthesize mannitol for fructose using a single enzyme absent from the less closely-related *T. gondii* [6].

Further progress will benefit from substantial research already devoted to these poultry parasites that cause an estimated annual global impact of $14B [59]. These poultry parasites also impose a significant burden to food safety by damaging the chicken gut, thereby increasing the risk of invasion by toxigenic *Clostridium perfringens*. The resulting condition, Necrotic Enteritis, elevates risk of human food poisoning with an agent whose spores can withstand 100 °C for more than one hour (FDA). For these reasons, poultry coccidiosis already receives major attention of public and private sector researchers. Established research procedures and foundational insights lend weight to adopting coccidiosis as a research model for *Cyclospora*.

**Researchers and animal caretakers can safely work with chickens and their parasites.** These poultry parasites pose no occupational health risk to researchers. This precludes the need to restrict experiments to high-cost, high-containment laboratories.

## 4. What Can We Learn from Such Surrogates?

Oocysts of *Eimeria acervulina* approximate the size of *C. cayetanensis*. This physical resemblance assists assessment of filtration and washing procedures. Other features of *E. acervulina* that make it a good surrogate include its prevalence, low infectious dose, high oocyst output and ease of use. It remains infectious at room temperature for long periods, giving it utility testing produce and other matrices. Mock contamination and treatment (radiation, washing, heat, pressure) has been attempted previously [60,61,62] with success. Therefore, the organism presents a readily available and safe alternative to study *Cyclospora.*

**Figure 2:**
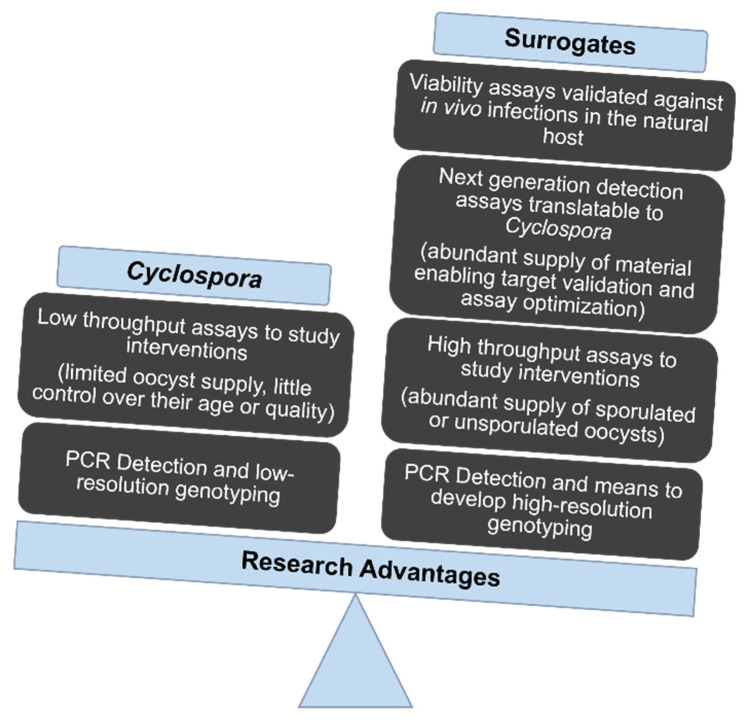
Opportunities to Study Surrogates of *Cyclospora cayetanensis*.

A single chicken can excrete millions of oocysts of *Eimeria*. Depending on the species of parasite, a single ingested oocyst can lead to the excretion of 1000–10,000 oocysts. Established protocols suspend or promote oocyst maturation in vitro, a process marked by known morphological transitions (sporulation = the development of sporocysts; excystation = the release of sporozoites from within those sporocysts). Optimized protocols for DNA and RNA extraction enable genomic and transcriptomic experiments. Bioinformatic resources include genome assemblies and annotation of various species of *Eimeria* and *Cyclospora*, who share many common features. Figure 2 demonstrates advantages of using *Eimeria* as a surrogate model for *C. cayetanensis*.

### 4.1. Detection

Existing methods to detect *C. cayetanensis* could be improved by evaluating and optimizing procedures using abundant and safe surrogates. Recent progress in DNA-based diagnostics provide exciting, newfound opportunities for surveillance and outbreak tracing. These include assays to amplify, from *C. cayetanensis*, portions of *18S rDNA* [54,63,64,65,66,67,68,69], apicoplast and mitochondrial DNA, and single copy variable nuclear markers suitable for source attribution [10,11,12,13,14,15,16,17,18,19,20]. Bacteriological Analytical Manual (BAM) protocols 19b [65,66,67,69,70,71] and 19c [72,73] accurately detect *C. cayetanensis* in various matrices, such as produce and water and are widely used and validated. A newly reported assay utilizes a mitochondrial target [74], further expanding the tools available for *C. cayetanensis* detection To date, multiple genomes of *C. cayetanensis* in public databases delimit our understanding of heritable variation in the species, providing a fund of additional markers capable of adding further epidemiological resolution, should that prove helpful.

The first published draft genome sequence for *C. cayetanensis*, reported by Qvarnstrom et al. (2015) [4] provided an assembly consisting of ~44 Mb, including a full-length mitochondrial genome of ~6.3 Kb and an apicoplast genome of ~24 Kb. Since then, advances in Next Generation Sequencing (NGS) technologies and bioinformatics have fostered additional draft genome assemblies, complete mitochondrial and apicoplast genomes of *C. cayetanensis* [3,5,6,7,8,9,10,57]. Several other studies have made strides on *C. cayetanensis* genomics and diagnostics, focusing on genotyping assays for use with stool and produce samples and molecular techniques to improve detection and sequencing (also cited above). Currently, 40 *C. cayetanensis* genome assemblies are deposited in the National Center for Biotechnology Information (NCBI); strain NF1_C8 [7] has been designated as the reference (RefSeq) strain.

Surrogates can accelerate further development of diagnostic and characterization tools by providing a safe, abundant source of organisms at defined stages of maturation and senescence, and over a range of concentrations and substrate complexities. Surrogates also provide meaningful controls to validate the specificity of molecular tools intended to exclusively detect *C. cayetanensis*. Importantly, by mixing *C. cayetanensis* with defined concentrations of *Eimeria*, these organisms provide the means to evaluate any assay’s performance in the presence of those organisms for which *Cyclospora* might most easily be confused.

Available diagnostic tools for *C. cayetanensis* require time, well-equipped laboratories, and well-trained laboratory personnel. The FDA BAM 19b method survived inter-laboratory validation [67], marking an important step forward for reference labs seeking to establish or rule out *Cyclospora* as a cause for enteric disease outbreaks. However, such research tools, though sensitive and specific, are time-consuming. Industry and regulators require real-time assessment of risk, requiring methods delivering inexpensive, immediate results.

Under conditions that exactly replicate the validated methods, this assay proved capable of discriminating its target from other, related parasites. However, recent attempts to adapt the method to surveillance purposes demonstrate that other parasites can generate amplicons bound by this probe; independent studies presented at the 2022 Center for Produce Safety Research Symposium, employing amplicon sequencing and phylogenetic analysis, suggest off-target amplification can occur (unpublished data). For this reason, more specific targets may further enhance surveillance goals. Indeed, a new mitochondrial target was recently developed [74] as a complementary tool to the BAM *18S rDNA* qPCR assay.

We are investigating simple sand and sand/zero valent iron filters for their ability to remove oocysts from surface water (evaluating their performance in water of varying turbidity), employing the abundance and safety of *Eimeria* surrogates to evaluate initial success. To leverage the untapped promise of other diagnostic approaches offering specificity, sensitivity, rapidity, and cost-sensitivity, surrogates:

- Provide an abundant and safe source of biological material necessary for establishing “proof of principle” and for optimizing assay conditions. For example, *Eimeria* oocysts may be well-suited to evaluating developmental pipelines for diagnostic aptamers [75,76] for inclusion in cheap, field-deployable ‘dip sticks.’
- Merit evaluation for their ability to trigger false positive test results. Cross-reactivity poses real risks for molecular diagnostic assays; because of their close evolutionary relationship and genetic similarity, *Eimeria* and *Cyclospora* can be easily mistaken for one another.

### 4.2. Maturation and Viability

Studying surrogates in their natural hosts circumvents the constraints impeding development of assays for viability and infectiousness in *C. cayetanensis*. Regulators seek such means, because the mere presence of parasite oocysts (or parasite DNA) need not necessarily endanger consumers. While the FDA has taken a major recent step forward by validating a method to detect oocyst contamination in food matrices, industry and regulators would benefit from means to determine the risk such oocysts pose to human health. Oocysts of *Eimeria* and *C. cayetanensis* initially lack the capacity to infect others when excreted. Oocysts become infectious after they have undergone maturation (sporulation) in the environment. For *Eimeria* spp., this process may take up to 48 h (range is 24–48 h). For *C. cayetanensis*, it is a longer process (below). Scientists have succeeded in describing this process for *Cyclospora* under defined conditions, but surrogates provide definitive means to discern the morphological and molecular changes that render oocysts infectious, and to evaluate how long they remain infectious under a variety of environmental conditions. We are performing long-term incubation studies to determine how long *Eimeria* oocysts remain viable, and to determine whether in vitro excystation (sporozoite release) predicts infectivity.. Directly studying that question in *Cyclospora* would require deliberately attempting to infect human subjects.

A hallmark of mature oocysts of both *Cyclospora* and *Eimeria* spp. are the double-layered, environmentally resistant sporocysts that enclose each pair of sporozoites. [51,77]. (Four such sporocysts occur in each oocyst of *Eimeria;* in *Cyclospora*, two such sporocysts occur. In the more distantly related *Toxoplasma gondii,* each of two sporocysts contain four sporozoites; more distantly related still, species of *Cryptosporidum,* lack sporocysts entirely- see Figure 1).

The unsporulated oocysts of *C. cayetanensis* are relatively uniform spheroids measuring 8–10 µm in diameter. Their thin (<1 µm), bilayered oocyst walls lack color. Unsporulated oocysts have a polar body and oocyst residuum [51]. In time, two sporocysts develop inside the oocysts. Each ovoid sporocyst, ~4 × 6 µm, contains both Stieda and substieda bodies and a large residuum. Within each sporocyst, two elongate sporozoites (~1 × 9 µm) develop [78]. Pairs of sporozoites in surrogate species of *Eimeria,* by contrast, form in each of four sporocysts.

The most relevant data on *Cyclospora* maturation come from studies of *Cyclospora* itself. No two parasite species mature according to precisely the same schedule, and distinct environmental conditions or signals may influence when they achieve infectiousness and for how long they remain infectious). Even among the species of *Eimeria* that infect chickens, differences exist, and all of them mature faster than *C. cayetanensis,* likely optimizing transmission among chickens. Nonetheless, surrogates add value by helping unpack what may be generally true of coccidian developmental biology, the general effectors of life history transitions, and the biomarkers predictive of infectiousness. In much the same way that pioneering insights relevant to human embryology ensued from studies of chicken, frogs, and zebrafish, development in *Eimeria* certainly parallels development in *Cyclospora*. In the words of statistician George E.P Box, “All models are wrong, but some are useful”.

As with surrogate species of *Eimeria,* ambient temperature influences the rate at which oocysts of *C. cayetanensis* mature. Under typical conditions, this takes a week or so. Under laboratory conditions, sporulation took 7–14 days when stored at 22 °C and 30 °C in deionized water or potassium dichromate [79]. Sporulation took only 4 days when oocysts were kept at 37 °C, and only 1 hr at 50 °C. Storage at 4 °C or 37 °C for 14 days retarded sporulation. Only 12% of human-and baboon-derived *Cyclospora* spp. sporulated. Oocysts, previously stored at 4 °C for one to two months, sporulated when later stored for 6 to 7 days at 30 °C.

Surrogates can broaden understanding of how sporulation rates vary, with temperature, in food matrices. Data exist for *C. cayetanensis* in dairy products and basil [80]. Sporulation occurred in those matrices at 23 °C, but not at extreme temperatures. No evident sporulation occurred at −70 °C, 70 °C, and 100 °C for either water or basil test samples; nor did oocysts sporulate when dairy products were cooked at 70 °C, frozen at −70 °C for 1 hr, or after exposure to −15 °C for 24 hr. Similarly, oocysts did not sporulate in basil kept stored at −20 °C for 2 days or in water for 4 days. Researchers performing these studies were handicapped by not being able to define the temporal interval between excretion and the tests. This limitation, superable using surrogates, warrants attention because prematurely expelled coccidian oocysts, in loose stools, may never sporulate.

Surrogate organisms offer an abundant source of material to evaluate broader conditions, characterize oocyst responses to a variety of environmental and chemical exposures, and identify biomarkers characteristic of immature, mature, senescent, and dead parasites. Orchestrated changes in gene expression characterize maturing oocysts of *E. acervulina* [81] and two-thirds of the genes undergoing the greatest changes have direct homologues in *C. cayetanensis*. We are evaluating gene expression under a variety of environmental conditions and leveraging such data to develop assays that predict viability and infectiousness. Importantly, we can test such predictions empirically by feeding such parasites to their natural hosts and assessing whether infection results.

### 4.3. Sanitation and Inactivation Treatments

What works best to rid produce, or the food processing environment, of viable oocysts of *Cyclospora*? To find out, no food processor would intentionally contaminate their facility with this dangerous and damaging parasite (even if sufficient oocysts could be marshalled for this purpose). Indeed, even our research facility modeling the produce production environment precludes experiments using harmful human pathogens. Surrogates provide a suitable solution, permitting informative experiments that pose no risk to the safety of laboratory personnel.

Such surrogates also hasten high-throughput screens for compounds capable of arresting parasite development. Coccidian oocysts in general are resistant to environmental influences and this resistance is derived from the biochemical makeup of inner layers of the oocyst and sporocysts [82,83]. Nothing is known of the biochemical composition of *Cyclospora* oocysts. However, *Toxoplasma* and *Eimeria* oocyst walls contain proteins, and a waxy coat of acid-fast lipids that makes them environmentally resistant [84,85]. Their environmental resistance is related to the highly elastic inner layer of the oocyst wall, because even the removal of outer layer by Clorox treatment does not affect the survival of sporulated oocyst. Recently, it has been reported that the inner layer of sporocyst wall is also environmentally resistant [86]. The environmental resistance afforded to species of *Cyclospora* and *Eimeria* cannot be investigated using species, such as *Cryptosporidium* spp., lacking sporocysts. In other respects, the body of knowledge acquired by working with *Eimeria*, *Toxoplasma,* and *Cryptosporidium* should promote new strategies to mitigate the removal of coccidian oocysts from irrigation waters.

Surrogates can help the produce industry evaluate the efficacy of those chemicals typically used for washing. These include chlorine-containing compounds (e.g., bleach, chlorine dioxide), acids (e.g., acetic, citric, peracetic, lactic), hydrogen peroxide, and ozone [87,88]. Unfortunately, coccidian oocysts notoriously resist free chlorine at levels far above those typical for drinking water. Therefore, studies on other coccidia (primarily using much more distantly related *Cryptosporidium parvum*) have focused on other disinfectants such as ozone [89,90], alternative chlorine-containing chemicals such as monochloramine [91], chlorine dioxide [92,93], and sequential treatment with ozone and then free chlorine [94] or monochloramine [95,96].

Arguably the best-studied responses to inactivation, for parasites in the coccidian family, is *Toxoplasma gondii* (reviewed in [97]). Although coccidian parasites can be inactivated by various means, they tolerate remarkable environmental extremes and remain infectious. Attempts to inactivate *Eimeria* spp., *T. gondii,* or *C. cayetanensis* with chlorine and ozone compounds showed only partial [98,99,100] or no effect [101,102]. *E. acervulina* oocysts, treated aggressively with the peroxygens 25% hydrogen peroxide and 5% peracetic acid for as long as 3-hrs, remained infectious [103]. However, oocysts exposed to hydrogen peroxide gas plasma were rendered non-infectious. Gaseous hydrogen peroxide is also effective at treating *C. parvum* oocysts [104]. A 10% dilution of 25% hydrogen peroxide and 5% peracetic acid combination was highly effective at reducing *C. parvum* oocyst sporulation and shedding in mice [105]. Another study showed that 30% hydrogen peroxide and 35% peracetic acid treatment from 1–30 min reduced *C. cayetanensis* sporulation by only 25%, although 5% hydrogen peroxide or 5% peracetic acid treatment for 1 min rendered *C. parvum* oocysts non-infectious (Doyle, unpublished data). These prior studies underscore the difficulty of using chemicals to render fresh produce safe, palatable, and free of viable coccidian oocysts. The evident variability among coccidian species argues for studying *Cyclospora* and its closest relatives, namely species if *Eimeria*.

*E. tenella* oocysts, refrigerated for 64–332 days, remained infectious (although recipient chickens excreted fewer oocysts and took longer to do so) [106]. Our own work, studying various species of *Eimeria*, affirms that oocysts stored for more than a year remain infectious. Although this presents obstacles for food sanitation, it provides an opportunity as a research model: properly stored parasites provide continuous means to evaluate the effects of radiation, heating, freezing, and high hydrostatic pressure [60,61,62]. We plan to conduct further studies on inactivation of oocysts in food matrices, aiming to replicate common agricultural conditions.

Surrogates also accelerate high-throughput screens for new compounds that might contribute to produce safety. A promising, ongoing effort uses image-analysis software trained to differentiate sporulated from unsporulated oocysts of *Eimeria* (Lenaghan, unpublished data). This tool facilitates automated, parallel screening of vast libraries of bioactive compounds. Limiting such screens to *C. cayetanensis* slowed progress because even sourcing developmentally synchronized oocysts (to train the image analysis software) proved difficult. Obtaining sufficient oocysts of *C. cayetanensis* to perform the screening proved more difficult, still. Screening many compounds against surrogates will focus attention on a subset of agents most likely to offer benefit in mitigating the risks posed by *Cyclospora*.

## 5. A Path to Future Progress

Although food safety professionals and regulators can detect DNA of *Cyclospora* in certain food matrices, they lack the means to determine whether viable parasites contaminate produce. True risk assessment requires viability assays. We see *Eimeria* surrogates as key to developing such tools, leveraging candidate biomarkers we have recently identified [81,107]. The first tools will likely remain in the purview of reference diagnostic labs. Eventually, produce producers and regulators require low-cost, field-deployable tools for surveillance, risk assessment, and risk mitigation.

For example, we are interested in exploring the power of DNA-binding dyes to differentiate live from dead parasites. For other pathogens, propidium monoazide (PMA) does not penetrate live cells but penetrates most dead ones. Thus, it can suppress amplification from free DNA or DNA residing in dead cells, enabling selective amplification from live (presumably viable) parasites. PMA has been utilized in PCR and qPCR assays for detection of viable protozoa [108,109,110,111], including the related parasites *Cryptosporidium* and *Toxoplasma*. Beginning with *Eimeria* surrogates and then with *C. cayetanensis*, researchers should establish whether dead oocysts are indeed fully permeable to the dye; otherwise, such assays might mistake dead for live parasites, overestimating food safety risk. Surrogates enable definitive evaluation of such a question in the natural host, without raising the ethical concerns of human subject studies.

Surrogates can also accelerate the evaluation of digital PCR to directly quantify nucleic acid targets, enabling absolute quantification and eliminating the dependency on standard curve estimates. Coupling this approach with staining methods, such as those described above, may provide diagnostic labs robust means to quantify genomes and transcripts from viable parasites. *Eimeria* surrogates constitute a safe and abundant resource for devising conducive and reproducible assay conditions. We are therefore commencing such work, seeking to quantify viable parasites amidst varying quantities of dead parasites and other contaminants. Surrogates also provide means to leverage the power of next-generation sequencing to characterize and mitigate the harms caused by *Cyclospora*. Comparative genomics has shown *C. cayetanensis* is related to *Eimeria* [3,5,6,58], and it possesses complete synteny in apicoplast and mitochondrial genomes [3]. Achieving draft genomes of *C. cayetanensis* has required research teams to overcome the challenges imposed by limited amounts of DNA; to date, several high-quality, publicly available genomes exist. Prior success entailed sequencing DNA derived from parasite cohorts excreted by symptomatic patients; far fewer oocysts can generally be isolated from water or food. Adding to the challenge, DNA resides within tough oocyst and sporocyst walls.

New techniques may greatly enrich the genotyping and genome sequencing, and surrogates provide the best means to evaluate and optimize these methods. For example, surrogates offer powerful means to evaluate methods that enriching target nucleic acids from complex mixtures. Capture enrichment sequencing (CES) greatly enriches pathogen genomes in a great range of biological contexts. Examples include capturing DNA from *Wolbachia* (a symbiont of insects and some nematodes) [112], *Yersinia pestis* DNA (from Black Death victims) [113], and *Leishmania* [114] genomes. Prior researchers achieved 1600-fold enrichment of target RNA of fungal transcripts [115]. If a sample’s DNA differs modestly from the known sequence, capture enrichment sequencing may succeed where PCR-based genome amplification methods fail. The approach uses long oligonucleotides (90–120 nt) as baits. These bind targets of ≥60 nt, so long as they are ≥80% similar. Thoughtful assay design allows researchers to target specific regions of the genome, whether ultra-conserved elements (UCEs) or fast-evolving mitochondrial DNA (mtDNA) or restriction-site associated DNA (RAD loci). Hence, this approach may provide means to genotype *Cyclospora* from produce or stool samples and further illuminate the population genetic structure at high resolution. We have used similar methods to selectively amplify *Cryptosporidium*. This approach may also furnish high-resolution genetic markers for mapping and functional analysis. Surrogates can be utilized to standardize single cell genomics methodology by sorting individual oocysts followed by amplifying whole-genome and sequencing [116] for revealing genome-level variation and mixed infection in clinical samples from *Cyclospora*-infected hosts.

Such studies could complement established methods such as Multi-locus Sequence Typing (MLST) [11,14,17,19,20,21] that have proven useful in source tracing and cluster investigations of *C. cayetanensis*. Additional innovation might provide more resolution to strengthen these efforts. Surrogates provide the ample genetic resource needed to optimize assays of this kind, providing the experimental basis to develop additional tools for elucidating the epidemiology of *C. cayetanensis*. They also provide source material applicable for developing nanotechnology-based biosensors.

The impact of cyclosporiasis mounts, requiring actionable information and effective management tools. Surrogates, we argue, hold great promise to accelerate progress. *C. cayetanensis* surfaces only episodically, causes injury even when scarce, poses risk to the health of researchers, and cannot yet be propagated in the laboratory. For all these reasons, researchers should leverage the benefits of safe, abundant, and renewable surrogates to address and ameliorate this growing threat.
